# Finding biological process modifications in cancer tissues by mining gene expression correlations

**DOI:** 10.1186/1471-2105-7-6

**Published:** 2006-01-09

**Authors:** Giacomo Gamberoni, Sergio Storari, Stefano Volinia

**Affiliations:** 1 ENDIF – Dipartimento di Ingegneria, Università di Ferrara, Ferrara, Italy; 2 Dipartimento di Morfologia ed Embriologia, Università di Ferrara, Ferrara, Italy; 3 DAMA – Data Mining for DNA Microarrays, Telethon Facility, Ferrara, Italy

## Abstract

**Background:**

Through the use of DNA microarrays it is now possible to obtain quantitative measurements of the expression of thousands of genes from a biological sample. This technology yields a global view of gene expression that can be used in several ways. Functional insight into expression profiles is routinely obtained by using Gene Ontology terms associated to the cellular genes. In this paper, we deal with functional data mining from expression profiles, proposing a novel approach that studies the correlations between genes and their relations to Gene Ontology (GO). By using this "functional correlations comparison" we explore all possible pairs of genes identifying the affected biological processes by analyzing in a pair-wise manner gene expression patterns and linking correlated pairs with Gene Ontology terms.

**Results:**

We apply here this "functional correlations comparison" approach to identify the existing correlations in hepatocarcinoma (161 microarray experiments) and to reveal functional differences between normal liver and cancer tissues. The number of well-correlated pairs in each GO term highlights several differences in genetic interactions between cancer and normal tissues. We performed a bootstrap analysis in order to compute false detection rates (FDR) and confidence limits.

**Conclusion:**

Experimental results show the main advantage of the applied method: it both picks up general and specific GO terms (in particular it shows a fine resolution in the specific GO terms). The results obtained by this novel method are highly coherent with the ones proposed by other cancer biology studies. But additionally they highlight the most specific and interesting GO terms helping the biologist to focus his/her studies on the most relevant biological processes.

## Background

From DNA microarray experiments, we can obtain genome-wide data about gene expression [1-3]. Each gene may be involved in one or more biological process/es. The biological process is described in the Gene Ontology datatabase (GO) provided by the GO consortium [4]. Merging microarray results, gene information and GO data within an experimental dataset allows efficient mining of functional knowledge and, for example, it can be useful in identifying differences between normal and cancer tissues.

Mutations are gained during carcinogenesis and tumour progression. Chromosomal rearrangements too lead to dysregulation of a number of cellular processes. We therefore hypothesized that it should be possible to identify deranged molecular pathways by mining expression profiles. The rationale is based on the assumption that although expression data do not give direct insight into mutations and rearrangements, they can reveal the molecular imprints consequential to oncogenic changes in cellular DNA. In fact, because tumours are the results of stratified genetic modifications, we reasoned that the normal cellular pathways of wild type cells should be affected in their balance of gene expression. Thus we designed and implemented a simple method for detecting such functional imbalances.

In this paper, we investigate an approach for studying the correlations between genes and their relations with Gene Ontology. The approach explores all possible pairs of genes, valuing the correlation between their expression, identifies the pairs with a correlation level higher than a threshold, and then relates these correlations to biological processes.

The approach is applied to a real dataset, represented by a gene expression matrix of hepatocellular carcinoma (HCC) [5-7]. This dataset collects the results of 161 microarray experiments (95 cancer samples, 66 normal liver samples). The results of our approach were compared to those of other approaches and it was observed that functional correlations comparison helps to identify meaningful information about the tissue behaviour. Applying this method to the hepatocarcinoma dataset for example it is possible to differentiate normal and cancer tissues and to identify those cellular processes and molecular functions which are deregulated during cancer establishment.

### The Gene Ontology

An ontology is a restricted structured vocabulary of terms that represent domain knowledge. In a practical sense, an ontology specifies a vocabulary that can be used to exchange queries and assertions. A commitment to the use of the ontology is an agreement to use the shared vocabulary in a consistent way. There is no commitment to completeness, the commitment is to coherence and consistency.

The Gene Ontology (GO) consortium produces three independent ontologies for gene products. The three ontologies form the basis for the description of the molecular function, biological process and cellular component of gene products. The relationships between gene products and specific terms in the three ontologies, molecular function, biological process and cellular component, are all many to many. In this work we focused only on the biological process terms, which should help to concisely describe the results of microarray experiments.

### Related work

In literature, there is a number of method for GO analysis.

GOAL (Gene Ontology Automated Lexicon) [8] is a web resource for automated and streamlined functional analysis of expression profiles. It aims to detect those GO terms which are significantly regulated. It automatically generates and evaluates scoring of GO terms from the results of an expression profiling experiment. Permutation analysis is performed to define P-values and false detection rates within each dataset.

Other related works that present some GO oriented analysis, are MAPPfinder [9], GoMiner [10] and EASE [11]. They introduce software packages designed to help biologists with the interpretation of genome-scale data.

MAPPfinder is an accessory program to GenMAPP, and is used to find the MAPP pathways most enriched for the genes in given gene list using a z-score metric.

GoMiner is a program for visualizing the genes on a list within the context of the structure of the Gene Ontology. Such an analysis leaves finding the most significant categories to visual inspections; that is, the user must manually scan the entire tree/DAG visualization to find the over-represented categories, and no correction is offered to address the multiple comparison problem.

EASE performs theme discovery with any list of genes. Theme discovery is defined as the identification of terms or phrases that describe a statistically significant number of genes in the list with respect to the number of genes described by the term or phrase in the population of genes from which the list derived.

These last three systems do not treat the gene expression information in any way, as they analyze only the statistical difference between two lists of genes. The approach we describe here, functional correlations comparison, is in principle fairly different, as: i) it is based on gene pairs rather than gene singlets, and ii) it measures correlation between expression levels, rather then differential expression. This novel method is also classification oriented, and it aims to highlight differences between pathologic and normal samples.

### Purpose

By linking the patterns of gene-pairs expression to the respective gene function (as provided by the Gene Ontology database), we can extract information to better understand genome wide expression profiles and to help scientists in the subsequent design of focused experiments. As a proof-of-principles, we identified the GO terms which distinguish cancer tissues from their normal counterparts. Since cancer cells vary heavily their patterns of gene regulation, we searched for the most variable correlations between genes involved in the same cellular processes. This technique should enable the identification of an additional layer of information to better comprehend the major biochemical and cellular steps followed during cancer establishment and progression.

In details, the functional correlations comparison aims to highlight changes in gene expression correlations, in order to identify relations that are lost or gained in cancer. Merging these results with the GO annotations, we can immediately select functionally relevant biological entities associated to pathogenic abnormalities.

We used the Pearson's correlation coefficient (described in "Methods" Section) to evaluate the correlation between the mRNA levels of all possible gene pairs. Finally, we linked these results with the GO terms and selected the significant functional differences.

This approach can be applied in two alternative ways: i) to all variable genes, i.e. by pre-selecting all genes that are reasonably variable in the expression matrix, irrespective of their association to disease or sample classification; ii) to the truly differentially expressed genes, so that only significant genes for sample classification are taken into account. The major feature of the functional correlations comparison being that it deals with gene pairs rather than singlets, and thus, unlike many supervised data mining techniques applied to transcriptome, it is geared towards measuring functional partnerships.

## Results

In the first step of our data analysis, we compute the correlation coefficient between the expression level for every possible pair of genes. More details about this process are described in "Methods" Section. We considered here the two datasets separately: normal tissue (LIV) and cancer (HCC).

In order to better manipulate the huge amount of experimental data, we defined and implemented a MySQL database [12]. All the measures were calculated using an Octave [13] script.

In order to identify the most significant correlations, we filtered the results, keeping only the gene pairs with the correlation coefficient above a threshold. Considering the absolute value of the correlation coefficient, we selected both positive and negative relations. The threshold was chosen in order to obtain an high significance level (at least of *p = *10^-9^) (the confidence limits were |*r*| ≥ 0.66657 for the LIV dataset and |*r*| ≥ 0.57617 for the HCC dataset).

We then obviously excluded from the results the correlations between different clones belonging to the same gene, as implemented by UniGene (i.e. by using the UniGene cluster definitions [14]).

In Table 1 and Table 2 we report the number of relations found in cancer and normal tissues (with a correlation coefficient above the threshold) for some GO terms (both genes in each pair must be linked to the same GO term). For a detailed view of all relation found, see Additional file 3 and Additional file 4.

**Table 1:** Correlations in HCC. In this table are listed some terms for which there is a significant number of relations in cancer but not in normal tissues. The table presents: in the first column, the name of the GO term; in the second (Pairs HCC) the number of correlations present in the cancer samples; in the third (Pairs LIV), the number of correlations found in the normal tissues. For each GO term, we report the mean and the standard deviation as computed by bootstrap analysis, as described in "Methods" Section.

**Gene Ontology term**	**Pairs HCC ***mean(stdev)*	**Pairs LIV ***mean(stdev)*
antigen presentation, endogenous antigen	2 0(0)	0 0(0)
blood coagulation	12 1.05(1.05)	2 1.3(1.3)
DNA replication	18 1.45(1.8)	2 1.45(1.4)
regulation of translational initiation	2 0.05(0.22)	1 0.25 (0.44)
DNA replication initiation	2 0.05(0.22)	0 0(0)
antigen processing, endogenous antigen via MHC class I	2 0.05(0.22)	0 0(0)
metabolism	33 9.45(3.4)	10 8.95(4.0)
cell cycle arrest	4 0.45(0.51)	0 1.15(2.0)
negative regulation of cell proliferation	10 1.95(1.3)	2 2.25(2.3)
chromatin remodeling	2 0.1(0.31)	0 0.15(0.37)
mitosis	6 0.7(0.86)	0 1(0.79)
heterophilic cell adhesion	3 0.15(0.49)	0 0.35(0.67)
cytokinesis	7 1.15(1.1)	0 1(1.1)
positive regulation of cell proliferation	4 0.7(0.66)	1 0.95(1.1)
cell adhesion	17 7.8(1.9)	3 11.4(5.6)
cholesterol metabolism	2 0.2(0.41)	0 0.25(0.72)
lipid transport	3 0.35(0.67)	0 0.45(0.69)
cell cycle	10 2.2(2.2)	5 2.3(2.2)
proteolysis and peptidolysis	14 4.15(2.9)	4 4.45(3.3)
protein complex assembly	5 0.95(1.3)	2 1.3(2.1)
antimicrobial humoral response (sensu Vertebrata)	3 0.3(0.92)	1 0.55(0.83)
cell motility	8 1.8(2.3)	4 1.65(1.6)
fatty acid metabolism	3 0.65(0.99)	1 0.55(0.94)

**Table 2:** Correlations in LIV. In this table are listed some terms for which there is a significant number of relations in normal tissues but not in cancer. The table presents: in the first column, the name of the GO term; in the second (Pairs HCC) the number of correlations present in the cancer samples; in the third (Pairs LIV), the number of correlations found in the normal tissues. For each GO term, we report the mean and the standard deviation as computed by bootstrap analysis, as described in "Methods" Section.

**Gene Ontology term**	**Pairs HCC ***mean(stdev)*	**Pairs LIV ***mean(stdev)*
intra-Golgi transport	0 0.1(0.31)	2 0.05(0.22)
ubiquitin-dependent protein catabolism	1 0.8(1.0)	4 0.35(0.49)
regulation of translation	0 0.2(0.70)	3 0.2(0.41)
glycolysis	1 0.15(0.49)	2 0.1(0.31)
electron transport	15 7.4(4.3)	35 9(4.6)
ER to Golgi transport	0 0.3(0.57)	4 0.35(0.67)
inactivation of MAPK	0 0(0)	2 0.15(0.37)
intracellular protein transport	23 18.1(7.8)	57 20.3(8.3)
small GTPase mediated signal transduction	4 1.35(1.5)	5 1.15(1.3)
mRNA processing	2 0.75(1.1)	4 0.8(1.1)
ubiquitin cycle	1 2.05(1.5)	10 2.3(2.7)
cell growth	1 0.3(0.57)	2 0.35(0.59)
N-linked glycosylation	0 0.45(0.94)	2 0.45(0.60)
regulation of cell cycle	3 2.35(1.5)	8 2.65(2.3)
DNA repair	1 0.85(0.88)	3 0.75(1.0)

In Table 1 and Table 2, and in the following discussion, we considered only Gene Ontology terms in which the number of gene pairs is significantly higher than that obtained during the simulation test (see "Methods" Section).

**Figure 1:**
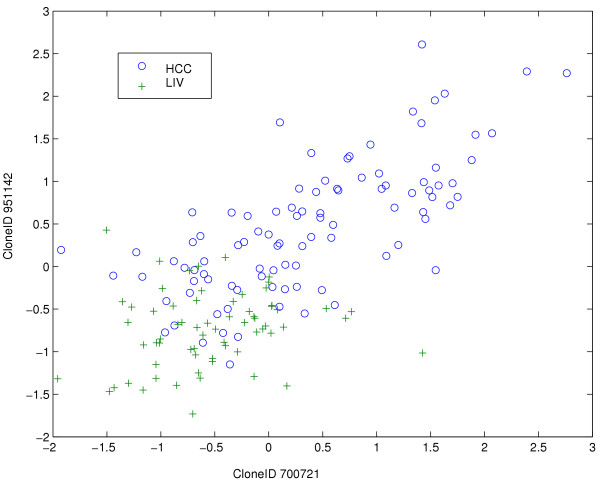
**Example of relation between two genes. **This is the expression profile for the clones 700721 (X axis) and 951142 (Y axis). Blue circles are the HCC samples, green crosses are LIV samples. We can see that the HCC have a good correlation (*r *= 0.71), while the LIV samples are not related (*r *= 0.17).

We assessed two different bootstrap approaches for the generation of the confidence limits (as described in "Methods" Section). In the first approach we randomly generated associations between genes and GO terms, while in the second we performed a bootstrap over sample classes, i.e. by randomly assigning class (HCC/LIV). A bootstrap analysis over HCC/LIV labels appeared though the least appropriate. For example, the behavior of the mRNA level could be that shown in Figure 1. In this case HCCs samples have a good correlation (*r *= 0.71), while the LIV samples do not (*r *= 0.17). Therefore by randomly assigning the HCC/LIV labels, we might obtain significant relations in both classes, and this would not be acceptable as a null hypothesis.

On the other hand, a simulation by random association of gene-GO terms proves to be relevant to our rationale. In fact the null hypothesis being that there is no relationship between independent gene pairs and their cellular function. Thus we used the simulation procedure where random association was performed between genes and GO terms.

Confidence limits were defined as the mean + 2 times the standard deviation gene pairs. I.e., we considered as significant a GO term associated with more than *mean *+ 2·*stdev *gene pairs (in a normal distribution, the corresponding one-tailed probability value is 0.02275).

There were 123 GO terms with at least one well correlated pair, although 36 GO terms were also identified in the simulation. Therefore we calculated a false detection rate (FDR) of 29% (36/123). Moreover, the results were simplified by retaining only the GO terms with more than one well correlated pair (51 left). We did not discuss the GO terms with significant numbers in both datasets, since these unaltered cellular components were not related to sample classification.

## Discussion

The GO terms we identified in HCC are very well in agreement with the over-expressed functions identified by Patil et al. [6]. The Gene Ontology terms selected here can be subdivided in two groups, according to their levels in the GO tree. When we considered the most generic functional entities, i.e those in the upper level of the tree, there was again good agreement between this analysis and that performed by other GO techniques. In addition, the functional correlations comparison identified a number of processes and functions in the lower part of the GO tree, where GO terms are more specific.

These specific GO terms at the bottom of the tree can be very useful for functional evaluation. They allow a more detailed patho-physiological dissection and enable a more focused molecular strategy for the experimental validation. Thus, it is apparent a higher resolution of this approach (a lower fraction of general GO terms are present in the results), at least in this experiment.

Furthermore, considering the results shown in Table 1 and Table 2, we obtained a fairly contained number of GO terms, highlighting the synthetic qualities inherent to the functional correlations comparison technique.

The gain of correlation pairs in cancer might be due to activation of gene networks absent in normal liver cells. The cancer tissues show many relations in the replication-related terms. *DNA replication *and *cell proliferation*, which appears in cancer, are of course basic processes in tumor expansion. Other highlighted terms are *cell adhesion, cell motility *and *cell cycle*, and all of these are usually involved in tumor development and metastasis.

On the other hand, all of the above mentioned GO terms do not present a significant number of relations in normal liver. Vice versa, other terms present more correlations in normal tissue, for example, *ubiquitin cycle *and *regulation of cell cycle*. Ubiquination of protein, a prominent process affecting regulation of cell cycle, is often involved in cancer. Lack of gene partners correlation in cancer might result from mutations leading to misexpression of the genes of these terms. The molecular mechanisms producing aberrant expression might be deletions, translocations, amplifications, or DNA methylation.

Summarizing, the results obtained by this novel method are highly coherent with the ones proposed by other cancer biology studies. Moreover they highlight the most specific and interesting GO terms helping the biologist to focus his/her studies on the most relevant biological processes.

## Conclusion

In the paper we proposed a novel approach to study the correlations between genes and their relations with Gene Ontology. This approach explores all possible pairs of genes, valuing the correlation between their expression, identifies the pairs with a correlation level higher than a threshold and then relates these correlations with biological processes. In this way we can identify and differentiate the functional relations between genes in normal tissue and cancer.

This approach was applied to a real dataset, represented by a gene expression matrix from hepatocellular carcinoma (including 95 cancer samples and 66 normal liver samples).

In this paper we described a method and performed some tests, showing some of its qualities: it identifies well defined differences between normal tissue and cancer; it provides very synthetic results; it identifies both generic and specific cellular processes.

Further studies will be conducted in other datasets in order to validate the applied methods and to test them together with their implementation in an appropriate software tool. Moreover, we will implement multiclass analysis, in order to better explore cancer complexity.

## Methods

### Experimental dataset

The analyzed dataset contains the results of 161 microarray experiments, of which 95 are HCC samples, and 66 normal liver samples. This experimental dataset was created by Chen et al. [5].

On the second channel of each experiment, there is a common reference RNA collection. So in the normalized gene expression matrix (7449 genes for 161 experiments) we can find the logarithm of the ratio of the two channel intensities. Sequences were identified by their IMAGE [15] CloneID.

As a filtering, the threshold for significant RNA expression changes (3.0-fold; i.e., 1.5 on the *log*_2 _scale) was established as three times the standard deviation (SD) of an assay, where the same RNA sample was independently retrotranscribed and labeled with both cyanines. DNA spots present in at least 75% of the arrays and with expression ratios higher than the above-defined threshold, in at least one array, were selected for the following analysis. The global median normalized dataset is available from the GOAL website [16], "Examples" section.

### Correlation study

In order to evaluate all the relations between clones, we studied any possible pair of clones in the dataset. If we consider expression level of *n *clones, we have n⋅(n−1)2
 MathType@MTEF@5@5@+=feaafiart1ev1aaatCvAUfKttLearuWrP9MDH5MBPbIqV92AaeXatLxBI9gBaebbnrfifHhDYfgasaacH8akY=wiFfYdH8Gipec8Eeeu0xXdbba9frFj0=OqFfea0dXdd9vqai=hGuQ8kuc9pgc9s8qqaq=dirpe0xb9q8qiLsFr0=vr0=vr0dc8meaabaqaciaacaGaaeqabaqabeGadaaakeaadaWcaaqaaiabd6gaUjabgwSixlabcIcaOiabd6gaUjabgkHiTiabigdaXiabcMcaPaqaaiabikdaYaaaaaa@3651@ pairs to study. In our case, with 7449 clones, we had to consider almost 28 × 10^6 ^possible pairs.

We studied all these possible relations, valuing the correlation coefficient of each pair. We applied this methods for two datasets, considering cancer and normal tissues separately. So, we can see relations differences between relations in these two type of cells.

### Correlation coefficient

The correlation between two variables reflects the degree to which the variables are related. The most common measure of correlation is the Pearson Product Moment Correlation (called Pearson's correlation for short). When computed in a sample, it is designated by the letter "*r*" and is sometimes called "Pearson's r". Pearson's correlation reflects the degree of linear relationship between two variables. It ranges from +1 to -1. A correlation of +1 means that there is a perfect positive linear relationship between variables. A correlation of -1 means that there is a perfect negative linear relationship between variables. A correlation of 0 means there is no linear relationship between the two variables.

This correlation is obtained using the following formula:

r=∑i=1N(xi−x¯)(yi−y¯)Nσxσy=     (1)
 MathType@MTEF@5@5@+=feaafiart1ev1aaatCvAUfKttLearuWrP9MDH5MBPbIqV92AaeXatLxBI9gBaebbnrfifHhDYfgasaacH8akY=wiFfYdH8Gipec8Eeeu0xXdbba9frFj0=OqFfea0dXdd9vqai=hGuQ8kuc9pgc9s8qqaq=dirpe0xb9q8qiLsFr0=vr0=vr0dc8meaabaqaciaacaGaaeqabaqabeGadaaakeaacqWGYbGCcqGH9aqpdaWcaaqaamaaqadabaWaaeWaaeaacqWG4baEdaWgaaWcbaGaemyAaKgabeaakiabgkHiTiqbdIha4zaaraaacaGLOaGaayzkaaaaleaacqWGPbqAcqGH9aqpcqaIXaqmaeaacqWGobGta0GaeyyeIuoakmaabmaabaGaemyEaK3aaSbaaSqaaiabdMgaPbqabaGccqGHsislcuWG5bqEgaqeaaGaayjkaiaawMcaaaqaaiabd6eaoHGaciab=n8aZnaaBaaaleaacqWG4baEaeqaaOGaeq4Wdm3aaSbaaSqaaiabdMha5bqabaaaaOGaeyypa0JaaCzcaiaaxMaadaqadaqaaiabigdaXaGaayjkaiaawMcaaaaa@50AC@

=∑i=1N(xi−x¯)(yi−y¯)∑i=1N(xi−x¯)2⋅∑i=1N(yi−y¯)2.     (2)
 MathType@MTEF@5@5@+=feaafiart1ev1aaatCvAUfKttLearuWrP9MDH5MBPbIqV92AaeXatLxBI9gBaebbnrfifHhDYfgasaacH8akY=wiFfYdH8Gipec8Eeeu0xXdbba9frFj0=OqFfea0dXdd9vqai=hGuQ8kuc9pgc9s8qqaq=dirpe0xb9q8qiLsFr0=vr0=vr0dc8meaabaqaciaacaGaaeqabaqabeGadaaakeaacqGH9aqpdaWcaaqaamaaqadabaWaaeWaaeaacqWG4baEdaWgaaWcbaGaemyAaKgabeaakiabgkHiTiqbdIha4zaaraaacaGLOaGaayzkaaaaleaacqWGPbqAcqGH9aqpcqaIXaqmaeaacqWGobGta0GaeyyeIuoakmaabmaabaGaemyEaK3aaSbaaSqaaiabdMgaPbqabaGccqGHsislcuWG5bqEgaqeaaGaayjkaiaawMcaaaqaamaakaaabaWaaabmaeaadaqadaqaaiabdIha4naaBaaaleaacqWGPbqAaeqaaOGaeyOeI0IafmiEaGNbaebaaiaawIcacaGLPaaaaSqaaiabdMgaPjabg2da9iabigdaXaqaaiabd6eaobqdcqGHris5aOWaaWbaaSqabeaacqaIYaGmaaGccqGHflY1daaeWaqaamaabmaabaGaemyEaK3aaSbaaSqaaiabdMgaPbqabaGccqGHsislcuWG5bqEgaqeaaGaayjkaiaawMcaamaaCaaaleqabaGaeGOmaidaaaqaaiabdMgaPjabg2da9iabigdaXaqaaiabd6eaobqdcqGHris5aaWcbeaaaaGccqGGUaGlcaWLjaGaaCzcamaabmaabaGaeGOmaidacaGLOaGaayzkaaaaaa@66D9@

Where *x*_*i *_and *y*_*i *_are the values of two variables on the i-th sample, x¯
 MathType@MTEF@5@5@+=feaafiart1ev1aaatCvAUfKttLearuWrP9MDH5MBPbIqV92AaeXatLxBI9gBaebbnrfifHhDYfgasaacH8akY=wiFfYdH8Gipec8Eeeu0xXdbba9frFj0=OqFfea0dXdd9vqai=hGuQ8kuc9pgc9s8qqaq=dirpe0xb9q8qiLsFr0=vr0=vr0dc8meaabaqaciaacaGaaeqabaqabeGadaaakeaacuWG4baEgaqeaaaa@2E3D@ and y¯
 MathType@MTEF@5@5@+=feaafiart1ev1aaatCvAUfKttLearuWrP9MDH5MBPbIqV92AaeXatLxBI9gBaebbnrfifHhDYfgasaacH8akY=wiFfYdH8Gipec8Eeeu0xXdbba9frFj0=OqFfea0dXdd9vqai=hGuQ8kuc9pgc9s8qqaq=dirpe0xb9q8qiLsFr0=vr0=vr0dc8meaabaqaciaacaGaaeqabaqabeGadaaakeaacuWG5bqEgaqeaaaa@2E3F@ the mean values of these variables, while *σ*_*x *_and *σ*_*y *_are their standard deviation.

### Significance level of Pearson's correlation

One tests the hypothesis that the correlation is zero (*r *= 0) using this formula:

t=r⋅n−21−r2     (3)
 MathType@MTEF@5@5@+=feaafiart1ev1aaatCvAUfKttLearuWrP9MDH5MBPbIqV92AaeXatLxBI9gBaebbnrfifHhDYfgasaacH8akY=wiFfYdH8Gipec8Eeeu0xXdbba9frFj0=OqFfea0dXdd9vqai=hGuQ8kuc9pgc9s8qqaq=dirpe0xb9q8qiLsFr0=vr0=vr0dc8meaabaqaciaacaGaaeqabaqabeGadaaakeaacqWG0baDcqGH9aqpcqWGYbGCcqGHflY1daGcaaqaamaalaaabaGaemOBa4MaeyOeI0IaeGOmaidabaGaeGymaeJaeyOeI0IaemOCai3aaWbaaSqabeaacqaIYaGmaaaaaaqabaGccaWLjaGaaCzcamaabmaabaGaeG4mamdacaGLOaGaayzkaaaaaa@3E72@

where *r *is the correlation coefficient and *n *is sample size, and where one looks up the *t *value in a table of the distribution of *t*, for *n *- 2 degrees of freedom. If the computed *t *value is as high or higher than the table *t *value, then the researcher concludes the correlation is significant (that is, significantly different from 0). So we can use the inverse equation, in order to find a threshold for filtering the *r *in order to guarantee a fixed level of significance:

r=t2t2+(n−2).     (4)
 MathType@MTEF@5@5@+=feaafiart1ev1aaatCvAUfKttLearuWrP9MDH5MBPbIqV92AaeXatLxBI9gBaebbnrfifHhDYfgasaacH8akY=wiFfYdH8Gipec8Eeeu0xXdbba9frFj0=OqFfea0dXdd9vqai=hGuQ8kuc9pgc9s8qqaq=dirpe0xb9q8qiLsFr0=vr0=vr0dc8meaabaqaciaacaGaaeqabaqabeGadaaakeaacqWGYbGCcqGH9aqpdaGcaaqaamaalaaabaGaemiDaq3aaWbaaSqabeaacqaIYaGmaaaakeaacqWG0baDdaahaaWcbeqaaiabikdaYaaakiabgUcaRiabcIcaOiabd6gaUjabgkHiTiabikdaYiabcMcaPaaacqGGUaGlaSqabaGccaWLjaGaaCzcamaabmaabaGaeGinaqdacaGLOaGaayzkaaaaaa@3F07@

Choosing a level of significance, we search *t *in the *t *table with *n *- 2 degrees of freedom and using Equation 4 we easily achieve the minimum *r *required for such confidence.

### Bootstrap analysis

We can ask if there are more relations between genes in one GO term than one might expect by chance. If that is true, then that term can be thought of as being overcorrelated in the data. This question can be answered comparing the obtained results with a null distribution.

In order to build this null distribution, we used a bootstrap method [17]. We performed 20 trials, shuffling the gene annotations each time. For each trial, we computed the number of pairs of genes associated to each GO term, and finally we computed its mean and standard deviation (*stdev*).

We also considered another bootstrap approach, based on random assignment of HCC/LIV labels. We performed 20 trials and computed, mean and standard deviation for the number of pairs of genes associated to each GO term. Further details are described in "Results" Section.

Complete results for both bootstrap analyses are reported in Additional file 1 and Additional file 2.

## Authors' contributions

SV provided the dataset, its description and every biological consideration. GG and SS implemented the algorithm, built the database and wrote the manuscript. All authors read and approved the final manuscript.

## Supplementary Material

Additional File 3 This .csv file contains data for Table 5 – Relations in HCC. In this table are reported all the well-correlated gene pairs found for each GO term in HCC samples. The table presents: in the first column, the name of the GO term; in the second and third column, the CloneIDs of the two correlated genes. Click here for file

Additional File 4 This .csv file contains data for Table 6 – Relations in LIV. In this table are reported all the well-correlated gene pairs found for each GO term in LIV samples. The table presents: in the first column, the name of the GO term; in the second and third column, the CloneIDs of the two correlated genes. Click here for file

Additional File 1 This .csv file contains data for Table 3 – Bootstrap with random GO term annotation. In this table are reported complete results for our first bootstrap analysis (shuffling GO term annotation). The table presents: in the first column, the name of the GO term; in the second (pairs HCC) the number of correlations present in the cancer samples; in the third (pairs LIV), the number of correlations found in the normal tissues; in the fourth and fifth column the mean and the standard deviation as computed by bootstrap analysis for HCC samples; in the sixth and seventh column, the mean and the standard deviation as computed by bootstrap analysis for LIV samples. Click here for file

Additional File 2 This .csv file contains data for Table 4 – Bootstrap with random HCC/LIV assignment. In this table are reported complete results for our second bootstrap analysis (random assignment to HCC/LIV class). The table presents: in the first column, the name of the GO term; in the second (pairs HCC) the number of correlations present in the cancer samples; in the third (pairs LIV), the number of correlations found in the normal tissues; in the fourth and fifth column the mean and the standard deviation as computed by bootstrap analysis for HCC samples; in the sixth and seventh column, the mean and the standard deviation as computed by bootstrap analysis for LIV samples. Click here for file
